# Molecular Mechanisms Responsible for Therapeutic Potential of Mesenchymal Stem Cell-Derived Secretome

**DOI:** 10.3390/cells8050467

**Published:** 2019-05-16

**Authors:** Carl Randall Harrell, Crissy Fellabaum, Nemanja Jovicic, Valentin Djonov, Nebojsa Arsenijevic, Vladislav Volarevic

**Affiliations:** 1 Regenerative Processing Plant, LLC, Palm Harbor, FL 34176, USA; dr.harrell@regenerativeplant.org (C.R.H.); crissy@regenerativeplant.org (C.F.); 2 Department for Microbiology and Immunology, Center for Molecular Medicine and Stem Cell Research, Faculty of Medical Sciences, University of Kragujevac, 69 Svetozara Markovica Street, 34000 Kragujevac, Serbia; nemanjajovicic.kg@gmail.com (N.J.); arne@medf.kg.ac.rs (N.A.); 3 Institute of Anatomy, University of Bern, Baltzerstrasse 2, 3012 Bern, Switzerland; valentin.djonov@ana.unibe.ch

**Keywords:** mesenchymal stem cells, secretome, therapy, inflammatory diseases, degenerative diseases

## Abstract

Mesenchymal stem cell (MSC)-sourced secretome, defined as the set of MSC-derived bioactive factors (soluble proteins, nucleic acids, lipids and extracellular vesicles), showed therapeutic effects similar to those observed after transplantation of MSCs. MSC-derived secretome may bypass many side effects of MSC-based therapy, including unwanted differentiation of engrafted MSCs. In contrast to MSCs which had to be expanded in culture to reach optimal cell number for transplantation, MSC-sourced secretome is immediately available for treatment of acute conditions, including fulminant hepatitis, cerebral ischemia and myocardial infarction. Additionally, MSC-derived secretome could be massively produced from commercially available cell lines avoiding invasive cell collection procedure. In this review article we emphasized molecular and cellular mechanisms that were responsible for beneficial effects of MSC-derived secretomes in the treatment of degenerative and inflammatory diseases of hepatobiliary, respiratory, musculoskeletal, gastrointestinal, cardiovascular and nervous system. Results obtained in a large number of studies suggested that administration of MSC-derived secretomes represents a new, cell-free therapeutic approach for attenuation of inflammatory and degenerative diseases. Therapeutic effects of MSC-sourced secretomes relied on their capacity to deliver genetic material, growth and immunomodulatory factors to the target cells enabling activation of anti-apoptotic and pro-survival pathways that resulted in tissue repair and regeneration.

## 1. Introduction

Many degenerative and inflammatory diseases are in the focus of stem cell-based research. Among different populations of stem cells, mesenchymal stem cells (MSCs) represent the most promising resource for the cell-based therapy of inflammatory and degenerative diseases on the ground of their multi-lineage differentiation potential, immuno-modulatory properties and pro-angiogenic characteristics [1,2,3,4,5,6]. MSCs spontaneously differentiate into osteoblasts, chondrocytes and adipocytes regulating normal turnover and homeostasis of adult mesenchymal tissues [7,8]. Importantly, MSCs have a differentiation potential broader than initially thought. Under strictly defined in vitro conditions, MSCs could generate cells of neuro-ectodermal and endodermal origin, including neuronal cells, hepatocytes, cardiomyocytes, alveolar and gut epithelial cells, representing new therapeutic agents in the regenerative medicine [9,10,11,12]. Moreover, MSCs regulate proliferation, activation and effector functions of immune cells (macrophages, dendritic cells (DCs), natural killer (NK) and natural killer T (NKT) cells, neutrophils, basophils, eosinophils, mast cells, T and B lymphocytes), indicating their therapeutic potential in the treatment of autoimmune and inflammatory diseases. Since MSCs produce pro-angiogenic factors and are capable to trans-differentiate into functional endothelial cells (ECs), these stem cells are considered ideal candidates for cell-based regeneration of ischemic tissues [5].

Despite these promising results, findings obtained in already conducted experimental and clinical studies pointed at several challenges which have to be addressed for safe and efficient clinical use of MSCs [1,2,3,4]. Safety issues regarding un-wanted differentiation of transplanted MSCs are still a matter of debate, especially in the long-term follow up. Encapsulated structures containing calcifications and ossifications were found in the infarcted areas of MSC-treated hearts [5]. Vision loss, detached retinas and intraocular bleeding were observed in three patients with macular degeneration after treatment with adipose tissue-derived MSCs (AT-MSCs) [1].

Several clinical trials indicated that an optimal number of transplanted MSCs should be clearly defined with an aim to find the right balance between safety and effectiveness of MSC-based therapy in term of their immunosuppressive properties [5]. Some of MSC-treated patients with idiopathic pulmonary fibrosis (IPF) developed infection and reported respiratory symptoms within a short time frame after MSC injection, indicating that MSC-based treatment resulted in excessive suppression of immune response in the injured lungs [2]. Similarly, increased number of respiratory and gastrointestinal infections were observed in patients with inflammatory bowel diseases (IBDs) who received immunosuppressive drugs just before MSC injection [3].

Autologous transplantation of MSCs is difficult to attempt on patients with fulminant diseases because of a long cell preparatory period and cell transplantation timing. Since MSCs lack expression of co-stimulatory molecules and major histocompatibility complex (MHC) class II proteins, they were considered hypo-immunogenic and, accordingly, were used in allogeneic transplantation studies [4]. Nevertheless, there are several obstacles for safe allogeneic transplantation of MSCs. Firstly, allogeneic MSCs express MHC class I molecules and are not completely invisible to the recipient’s immune system. Therefore, after transplantation, MSCs could trigger allogeneic immune responses and provoke aggravation of on-going inflammation [5]. Next, MSCs are permissive for cytomegalovirus (CMV) and herpes simplex virus (HSV) infections and, accordingly, MSC allotransplants carry the risk of viral transmission to the recipients. Accordingly, MSCs must be screened for CMV and HSV in order to prevent viral infections in immunosuppressed patients [6].

Results obtained in a large number of experimental studies demonstrated that MSC-sourced secretome showed therapeutic effects similar to those observed after transplantation of MSCs [13,14,15,16,17,18,19,20,21,22,23,24,25,26,27,28,29,30,31,32,33,34,35,36,37,38,39,40,41]. Therefore, in this review article we summarized findings obtained in preclinical and clinical studies that delineated molecular and cellular mechanisms which were responsible for beneficial effects of MSC-derived secretomes in attenuation of degenerative and inflammatory diseases of hepatobiliary, respiratory, musculoskeletal, gastrointestinal, cardiovascular and nervous system. An extensive literature review was carried out in March 2019 across several databases (MEDLINE, EMBASE, Google Scholar, ClinicalTrials.gov), from 1990 to present. Keywords used in the selection were: “mesenchymal stem cells (MSCs)”, “secretome”, “extracellular vesicles (EVs)”, “exosomes (Exos)”, “acute liver failure (ALF)”, “liver fibrosis”, “inflammatory lung diseases”, “acute lung injury (ALI)”, “chronic obstructive pulmonary diseases (COPD)”, “asthma”, “idiopathic pulmonary fibrosis (IPF)”, “osteoarthritis”, “inflammatory bowel diseases (IBDs)”, “ulcerative colitis (UC)”, “Crohn’s disease (CD)”, “cardiovascular diseases”, “myocardial infarction””, “corneal injury”, “dry eye syndrome”, “glaucoma”, “ischemic brain injury”, “spinal cord injury”. All journals were considered, and an initial search retrieved 1578 articles. The abstracts of all these articles were subsequently reviewed by three of the authors (CRH, CF and VV) to check their relevance to the subject of this manuscript. Eligible studies had to delineate molecular and cellular mechanisms involved in the beneficial effects of MSC-derived secretomes, and their findings were analyzed in this review.

## 2. MSC-Derived Secretome as New, Cell-Free Therapeutic Agent in Regenerative Medicine

MSC-sourced secretome is defined as the set of MSC-derived bioactive factors (soluble proteins, nucleic acids, lipids and extracellular vesicles (EVs)) secreted to the extracellular space [37,40]. MSC–derived secretome consists of a soluble component and MSC-sourced encapsulated EVs [40,41]. MSC–derived EV contains a lipid bilayer enriched in proteins (tetraspanins, integrins, ligands for cell surface receptors) enabling trafficking, adhesion and endocrine effects of EV [37]. A large number of MSC-derived bioactive molecules including genetic materials (DNA, RNA fragments, microRNAs (miRNAs)), enzymes, signaling and signal transduction proteins, immunomodulatory and growth factors are enveloped by bilayer membrane [40].

MSC-sourced EVs encompass apoptotic bodies, microvesicles and exosomes (Exos), distinguishable by their size and origin in the cell [40,41]. Apoptotic bodies represent the biggest EVs (>1000 nm) which disintegrate from the MSCs during apoptosis. MSC-derived microvesicles are nino-sized (100–1000 nm) EVs that develop by budding from the plasma membrane [40]. Exos are the smallest MSC-sourced EVs (30–200 nm) that originate via the inward budding of the late endosome membranes called multivesicular bodies (MVBs). Upon the fusion of MVBs with the plasma membrane, MSC-derived Exos are released into the extracellular milieu where they exert their biological effects by modulating multiple cell signaling pathways in target cells [41]. It should be emphasized that MSC-derived conditioned medium (MSC-CM) contains the complete milieu of MSC-sourced soluble factors and vesicular elements [40,41]. Thus, MSC-Exo represents, in fact, a sub-population of EVs that is a part of the MSC-CM. However, the soluble component of MSC-CM may be separated from the microvesicle fraction by centrifugation, filtration, polymer precipitation-based methodologies, ion exchange chromatography and size-exclusion chromatography [40,41].

Importantly, MSC-derived Exos and microvesicles have overlapping size ranges and, therefore, methods currently employed to separate these two sub-populations of EVs had varying degrees of success. Accordingly, when separation could not be completely ascertained, these two MSC-sourced encapsulated products were collectively designated as MSC-derived EVs. On the contrary, when researchers managed to successfully isolate, characterize and separate Exos from other MSC-derived EVs, beneficial effects of MSC-Exos were highlighted and emphasized [40,41].

MSC-derived EVs may be carried to distant sites via biological fluids where, in endocrine manner, modulate function of immune cells, ECs, pericytes and other tissue-resident cells [37]. MSC-sourced EVs interact with target cells by different mechanisms. MSC-EVs bind to membrane-bound receptors and trigger intracellular signaling which enables internalization of their content in the target cells. Alternatively, MSC-EVs may fuse with the plasma membrane and deliver their content to the cytosol of target cell directly [40,41].

Various biological effects were observed in experimental animals after administration of MSC-CM and MSC-EVs. Importantly, MSC-sourced secretome bypassed many limitations of MSC-based therapy, including unwanted differentiation and potential activation of allogeneic immune response. MSC-derived secretome is practical for clinical use since it could be massively produced from commercially available cell lines avoiding invasive cell collection procedure [38]. In contrast to MSCs which had to be expanded in culture to reach optimal cell number for transplantation, MSC-sourced secretome is immediately available for treatment of acute conditions including fulminant hepatitis, cerebral ischemia and myocardial infarction [39]. Additionally, MSC-derived secretome provides convenient source of bioactive factors since its content may be evaluated in a manner analogous to conventional pharmaceutical agents [37]. As a result of these biological and logistical advantages over MSC-based therapy, administration of MSC-derived secretomes has been considered as a new, cell-free therapeutic approach for the treatment of inflammatory and degenerative diseases [13,14,15,16,17,18,19,20,21,22,23,24,25,26,27,28,29,30,31,32,33,34,35,36,37,38,39,40,41].

## 3. Molecular Mechanisms Responsible for Beneficial Effects of MSC-Derived Secretome

Both EVs and soluble component of MSC-CM were capable to promote tissue regeneration, suppress detrimental immune response and induce neo-angiogenesis in ischemic tissues [40,41]. Therefore, MSC-sourced secretomes showed immunoregulatory, angiomodulatory and anti-apoptotic effects that resulted in enhanced tissue repair and regeneration [13,14,15,16,17,18,19,20,21,22,23,24,25,26,27,28,29,30,31,32,33,34,35,36,37,38,39,40,41].

### 3.1. Immunomodulatory Properties of MSC-Derived Secretome

MSC-CM and MSC-Exos contain several immunomodulatory factors including transforming growth factor-β (TGF-β), hepatic growth factor (HGF), indolamine 2,3-dioxygenase-1 (IDO-1), interleukin (IL)-10, IL-1 receptor antagonist (IL-1Ra) and prostaglandin E2 (PGE2) [5,37,40,41]. By delivering TGF-β, MSC-CM and MSC-Exos attenuated IL-2-induced proliferation of CD4+T helper and cytotoxic CD8+ T lymphocytes by causing G1 cell cycle arrest in Jak-1/Stat-5 dependent manner [42,43]. MSC-sourced secretome contains thrombospondin 1 (TSP1) which regulates activation of TGF-β/Smad signaling. Accordingly, by delivering TSP1, MSC-Exos may induce suppression of TGF-β/Smad2/3 signaling and alleviate proliferation and cytotoxic potential of NK cells [44]. HGF acts synergistically with TGF-β and, therefore, HGF-containing MSC-CM and MSC-Exos suppress expansion of activated peripheral blood mononuclear cells (PB-MNCs) by inducing their apoptosis [43,44,45].

Similarly, by providing IDO-1, which degrades tryptophan to immunosuppressive Kynurenine (KYN), MSC-CM and MSC-Exos inhibited proliferation of PB-MNC, particularly activated T lymphocytes [5,43,46]. Administration of MSC-CM managed to inhibit generation of inflammatory Th1 and Th17 cells by causing G1 cell cycle arrest of naïve T-bet and RORγT-expressing T cells and by inducing tolerogenic and regulatory phenotype in DCs within peripheral lymph organs [5,43]. Regulatory DCs of MSC-CM or MSC-Exo-treated animals, in turn, through the production of KYN, induced expansion of CD4+CD25+FoxP3+ T regulatory cells (Tregs) enabling generation of immunosuppressive microenvironment in inflamed tissues [5]. Moreover, MSC-CM and MSC-Exos, in IDO-1/KYN-dependent manner, prevented conversion of immunosuppressive Tregs in inflammatory Th1/Th17 cells [47]. During activation of T cell receptor, signals from protein kinase B (PKB/Akt) and mammalian target of rapamycin (mTOR) caused reprogramming of Tregs into inflammatory IFN-γ and IL-17 producing CD4+Th1/Th17 cells [48]. MSC-Exos in IDO1/KYN-dependent manner, induced activation of general control nonderepressible 2 (GCN2) kinase which inhibited Akt/mTOR signaling in Tregs preventing their transdifferention in Th1/Th17 cells [48]. In line with these findings, significant increase in total number of IL-10 and TGF-β-producing Tregs were observed in MSC-CM or MSC-Exo-treated activated PB-MNCs as well as in injured tissues and peripheral lymph organs of MSC-Exo-treated mice with autoimmune and chronic inflammatory diseases (multiple sclerosis, inflammatory bowel disease (IBD) and type 1 diabetes mellitus) [49,50,51,52,53,54].

Balance between immunosuppressive Tregs and inflammatory Th17 cells was also regulated by sphingosine 1-phosphate (S1P)-containing MSC-Exos [55]. By delivering S1P to CD4+T cells, MSC-Exos promoted generation of FoxP3-expressing and IL-10-producing Tregs but attenuated expansion of inflammatory, IL-17-producing Th17 cells in peripheral blood of patients suffering from aplastic anemia. In an analogy, significantly increased Tregs/Th17 cell ratio and alleviated disease were observed in MSC-Exo-treated mice with aplastic anemia. Importantly, MSC-Exo-mediated beneficial effects were completely diminished when S1P signaling was inhibited [55].

IL-1Ra-bearing MSC-Exos were crucially important for the attenuation of skin inflammation and accelerated wound healing [56]. IL-1Ra is an immunosuppressive cytokine which, by competitive inhibition, prevents binding of inflammatory IL-1β to its receptor (IL-1 receptor (IL-1R)) [57]. In this manner, pro-inflammatory events initiated by IL-1:IL-1R binding, including enhanced expression of E-and P-selectins on ECs and consequent increased influx of circulating leucocytes in inflamed tissues, are inhibited by IL-1Ra [58]. It was recently revealed that production of IL-1Ra and its delivery by MSC-Exos is regulated by local concentration of inflammatory cytokines, particularly tumor necrosis factor alpha (TNF-α) [56]. TNF-α induces activation of nuclear factor κB (NF-κB) which results in up-regulation of Fas-associated phosphatase-1 (Fap-1) and caveolin-1 (Cav-1) in MSCs. Fas binds to Fap-1 and Cav-1 and activates Soluble N-ethylmaleimide-sensitive factor (NSF) Attachment protein Receptor (SNARE)-mediated membrane fusion resulting in enhanced secretion of IL-1Ra-bearing Exos in extracellular space. In this manner, TNF-alpha-primed MSCs, through the delivery of IL-1Ra-containing MSC-Exos, inhibit IL-1β:IL-1R signaling and protect tissues from inflammation-induced injuries [56].

In line with these findings, it was recently revealed that MSCs exposed to inflammatory cytokines (TNF-α and IFN-γ) generate MSC-CM and MSC-Exos with enhanced immunomodulatory properties [59]. IFN-γ and TNF-α provoke MSCs to express inducible nitric oxide synthase (iNOS) which increases IDO-1 activity in MSCs. Accordingly, administration of MSC-CM, in iNOS and IDO-1/KYN-dependent manner suppressed inflammatory and cytotoxic potential of T lymphocytes and NKT cells [5].

In line with these findings, PB-MNCs which were cultured in the presence of TNF-α and IFN-γ-stimulated MSC-Exos, produced lower amounts of 34 inflammation-related cytokines and chemokines, but significantly increased secretion of several anti-inflammatory mediators, including IL-10 [59]. IL-10 inhibits maturation, down-regulates expression of co-stimulatory molecules and attenuates antigen-presenting function of DCs which results in suppression of T cell-driven inflammation [60]. TNF-α and IFN-γ-priming significantly increased concentration of PGE2 in MSC-CM and MSCs-Exos [59]. PGE2 has an important role in immunosuppression mediated by MSC-derived secretomes [43,60]. PGE2 has direct inhibitory effects on IL-2 production and attenuates expression of janus kinase (Jak)-3 which mediates the responsiveness of T cells to IL-2 [61]. Accordingly, MSC-CM and MSCs-Exos, in a PGE2-dependent manner, suppressed clonal expansion of activated T cells and attenuated T cell-driven inflammation [5,60,62]. Additionally, through the secretion of PGE2, MSC-sourced CM and Exos favored alternative activation of macrophages, prevented maturation of DCs and suppressed cytotoxicity of NK and NKT cells [5]. NK and NKT cells, cultured in the presence of PGE2-containing MSC-CM, failed to optimally express cytotoxic molecules and significantly reduced production of inflammatory cytokines (TNF-α, IFN-γ and IL-17) upon activation [5,43].

An enhanced immunosuppressive property of TNF-α and IFN-γ-primed MSC-Exos are in line with previously published data showing that MSCs have a dynamic response to local microenvironment [5]. As far as we know to date, MSCs are not constitutively immunosuppressive. They alter their secretory profile and immunomodulatory characteristics in dependence of inflammatory milieu to which they are exposed. In the presence of low concentration of IFN-γ and TNF-α, MSCs obtain pro-inflammatory phenotype and produce large amounts of inflammatory cytokines and chemokines that stimulate activation and migration of immune cells in inflamed tissues. On the contrary, when MSCs are exposed to the high levels of inflammatory cytokines, they adopt anti-inflammatory phenotype and secrete immunosuppressive factors that inhibit generation of inflammatory M1 macrophages, maturation and antigen-presenting function of DCs, effector functions of inflammatory CD4+Th1, CD4+Th17 cells, CD8+ cytotoxic T lymphocytes (CTLs), NK and NKT cells [5]. In line with these findings, IFN-γ and TNF-α-priming of MSCs should be used to promote generation of MSC-Exos with enhanced immunosuppressive properties that could have better therapeutic effects in the treatment of autoimmune and inflammatory diseases.

### 3.2. The Role of MSC-Sourced Secretome in Tissue Repair and Regeneration 

In addition to immunosuppressive cytokines, MSC-sourced secretome also contains cocktail of growth factors which promote tissue repair and regeneration, wound healing and neo-angiogenesis. Elevated concentration of tissue inhibitors of metalloproteinase (TIMP)-1 and 2, fibroblast growth factor (FGF)-6 and 7 and HGF were considered responsible for beneficial effects of MSC-CM in corneal epithelial wound healing [63]. Similarly, HGF-containing MSC-CM was involved in liver repair, regeneration [64], MSC-derived neurotrophins (brain-derived neurotrophic factor (BDNF) and nerve growth factor (NGF)), which were crucially important for MSC-CM-based alleviation of spinal cord injury [65].

MSC-CM-induced anti-fibrotic and angiomodulatory effects were also responsible for enhanced wound healing and reduced scar formation in MSC-CM treated animals [37]. Concentration of pro- and anti-angiogenic factors in MSC-CM is regulated by inflammatory and hypoxic conditions to which MSCs were exposed. When MSCs are cultured in the presence of high concentration of inflammatory cytokines and engraft in the inflammatory microenvironment, they start to produce anti-angiogenic molecules in order to prevent migration of circulating leucocytes in the inflamed tissues [37]. An extensive proteomic analysis of MSC-CM revealed that MSC-derived TIMP-1 was mainly responsible for anti-angiogenic effects of MSC-sourced secretome [66].

Hypoxia induces enhanced production of hypoxia-inducible factor 1 alpha (HIF-1α), which interacts with autophagy-related mitogenic neuropeptide Apelin and promotes survival and proliferation of MSCs [67]. Accordingly, HIF-1α has been considered a master regulator of proliferative and angiomodulatory function of MSCs when these stem cells are cultured under hypoxic conditions [68]. MSC-derived HIF-1α induces increased production of vascular endothelial cell growth factor (VEGF) and has crucially important role for pro-angiogenic effects of vascular endothelial growth factor (VEGF)-containing MSC-Exos [69]. Capacity of MSC-Exos to promote VEGF-dependent blood vessel formation during bone regeneration was completely abrogated by HIF-1α inhibitor [69]. In addition to VEGF, MSC-derived secretome contains several other pro-angiogenic factors (basic fibroblast growth factor (bFGF), TGF-β, platelet-derived growth factor (PDGF), angiopoietin-1, placental growth factor (PGF), IL-6, monocyte chemotactic protein-1 (MCP-1), epidermal growth factor (EGF), HGF) which showed beneficial effects in MSC-CM-based therapy of ischemic diseases [70].

Several recently published studies revealed that MSC-derived secretome was also able to regulate apoptosis in physiological and pathological conditions. MSC-CM-based therapy significantly decreased expression of pro-apoptotic Bax and cleaved caspase-3 but increased expression of anti-apoptotic Bcl-2 in parenchymal cells, preventing their loss during on-going inflammation [37]. Interestingly, completely opposite effects were noticed in MSC-CM-treated tumor cells. Significantly increased activity of caspase-3, -8, -9, -12 were noticed in MSC-CM-treated MDA-MB-231 breast cancer cells [71]. Importantly, these findings were also confirmed in vivo, in a xenograft mouse tumor model. MSC-CM treatment resulted in significantly reduced breast cancer growth and increased survival of tumor-bearing mice [71]. However, it should be noted that these beneficial effects were only observed in tumor bearing mice that received human uterine cervical MSC-derived conditioned medium (hUTC-MSC-CM) while treatment with human adipose tissue MSC-derived conditioned medium (hAT-MSC-CM) did not result in attenuated tumor growth. Compared to hAT-MSC-CM, hUCT-MSC-CM contains high levels of factors which induce apoptosis of tumor cells (tumor necrosis factor superfamily member 14 (TNFSF14) and promote anti-tumor Th1 cell-mediated immune response (C-X-C motif chemokine ligand (CXCL)10 and Fms-related tyrosine kinase 3 ligand) [37]. On the other hand, umbilical cord (UCD)-MSC-CM contains low levels of factors which promote tumor growth (epidermal growth factor receptor (EGFR), FGF-4 and -9), neo-angiogenesis (VEGF, IL-6, IL6 receptor), homing of naïve T cells to peripheral lymph nodes (chemokine (C-C motif) ligand 7 (CCL7)) and migration of circulating monocytes in tumor tissue (macrophage migration inhibitory factor (MMIF)) [37]. These findings indicate that MSC-CM-based effects depend on MSCs origin and suggest that content of MSC-CM should be precisely determined before clinical application.

## 4. Experimental Evidence for Therapeutic Potential of MSC-Derived Secretome in the Treatment of Inflammatory and Degenerative Diseases

A large number of experimental studies explored therapeutic potential of MSC-sourced secretome and their findings indicated that MSC-CM and MSC-Exos managed to efficiently enhance endogenous healing process in inflamed tissues by providing pro-angiogenic and trophic factors to injured cells, and by suppressing detrimental local and systemic immune response [13,14,15,16,17,18,19,20,21,22,23,24,25,26,27,28,29,30,31,32,33,34,35,36,37,38,39,40,41]. MSC–derived secretome showed beneficial effects in the treatment of inflammatory and degenerative diseases of hepatobiliary, respiratory, skeletal, gastrointestinal, cardiovascular and nervous system [13,14,15,16,17,18,19,20,21,22,23,24,25,26,27,28,29,30,31,32,33,34,35,36,37,38,39,40,41].

### 4.1. Beneficial Effects of MSC-Derived Secretome in the Treatment of Acute Liver Failure and Liver Fibrosis

Accumulating evidence suggest that MSC-sourced CM and Exos may represent a compelling alternative to MSCs in the treatment of ALF and liver fibrosis (Table 1) [13,14,15,16,17,18,72,73,74,75,76,77,78,79,80,81,82,83,84,85,86,87,88].

By using several animal models of ALF, we and others demonstrated that administration of MSC-CM significantly improved liver regeneration and increased survival rate of experimental animals by reducing influx of inflammatory cells in the inflamed liver and by attenuating apoptosis and increasing proliferation of injured hepatocytes [15,16,17,72,73,74,75,76]. Importantly, therapeutic effects of MSC-CM were similar to those observed after transplantation of their parental MSCs [74,75,76]. Monitoring of adoptively transferred leukocytes revealed that a reduced influx of circulating immune cells in injured livers was a consequence of MSC-CM-mediated down-regulation of chemokine receptors (CXCR3 and CCR5), responsible for the trafficking of IFN-γ and IL-17-producing inflammatory T cells [15,75]. Among various numbers of MSC-derived immunomodulatory, trophic and hepatoprotective factors, MSC-CM-induced prevention of apoptosis and enhanced regeneration of injured hepatocytes was mainly mediated by IDO-1/KYN, HGF, fibrinogen-like protein 1 and IL-6/gp130 signaling pathways [15,16,74,76].

In line with these findings, we recently demonstrated that bone marrow (BM)-MSC-CM, in an IDO-1/KYN-dependent manner, efficiently alleviated ALF in mice by suppressing pro-inflammatory and cytotoxic potential of liver NKT cells, the main effector cells in fulminant hepatitis [74,76]. Attenuated expression of apoptosis-inducing ligands was observed on MSC-CM-treated liver NKT cells and was accompanied with reduced cytotoxicity of NKT cells against hepatocytes in vitro and in vivo. Additionally, MSC-CM-treated liver NKT cells had reduced capacity for production of inflammatory cytokines (TNF-α, IFN-γ, IL-4) and secreted higher amount of immunosuppressive IL-10 [76]. An addition of 1-methyl-dl-tryptophan (1-MT), a specific IDO-1 inhibitor, or l-N^G^-monomethyl arginine citrate, a specific inhibitor of inducible nitric oxide synthase (iNOS), completely abrogated immunosuppressive and hepatoprotective effects of MSC-CM and restored hepatotoxicity of NKT cells, suggesting that MSC-CM-mediated suppression of NKT cells was iNOS and IDO-1-dependent [76]. Having in mind that MSC-derived IDO-1 prevents trans-differentiation of Tregs in Th17 cells, we analyzed capacity of MSC-CM to regulate ratio between FoxP3-expressing, IL-10 producing immunosuppressive NKTreg cells and RORγT-expressing, IL-17-producing inflammatory NKT17 cells in the liver. Systemic administration of MSC-CM significantly reduced the total number of liver-infiltrating NKT17 cells and promoted expansion of liver NKTregs which resulted in attenuation of ALF [74]. Therapeutic potential of MSC-CM was completely abrogated by 1-MT, confirming the crucial importance of IDO-1/KYN pathway for immunosuppressive and hepatoprotective effects of MSC-CM in modulation of ALF [76].

In a similar manner as MSC-CM, administration of MSC-Exos prevented detrimental immune response and apoptosis of hepatocytes in ALF and inhibited production of TGF-β in HSCs, resulting in attenuation of liver fibrosis [13,77,78,79,80]. By using drug-induced models of ALF, Tan and coworkers showed that MSCs-Exos elicited hepatoprotective effects by enhancing expression of the anti-apoptotic gene Bcl-xL in injured hepatocytes and by promoting hepatocyte proliferation in Cyclin D1-dependent manner [81]. Additionally, Lou and associates revealed that MSC-Exos inhibited activation of inflammasome and production of inflammatory cytokines, (TNF-α, IFN-γ, IL-1β, IL-6 and IL-18) in liver-infiltrating leukocytes, indicating immunosuppressive effects of MSC-Exos in alleviation of ALF [13].

Administration of MSC-CM managed to alleviate liver fibrosis by suppressing activation of hepatic stellate cells (HSCs), the major source of extracellular matrix proteins in the liver [77,78,79]. In line with these findings are our results obtained in MSC-CM-mediated alleviation of liver fibrosis [18]. Injection of MSC-CM attenuated carbon tetrachloride (CCl4)-induced fibrosis in mice due to the IDO-1-dependent expansion of IL-10 producing Tregs and through the suppression of inflammatory IL-17-producing Th17 cells in fibrotic livers [18]. Lack of IL-17 led to the reduce production of collagen-1, α-smooth muscle actin and other pro-fibrotic molecules in HSCs [77,78]. Interestingly, MSCs produce IL-10 as a response to enhanced secretion of HSC-derived pro-fibrotic factors, indicating the importance of MSCs:HSCs cross-talk for MSC-Exo-based attenuation of liver fibrosis [14].

In addition to IL-10, NGF also had important role in MSC-CM-induced suppression of HSCs [79]. Activated HSCs express p75 receptor, which, upon NGF stimulation, triggers apoptosis in these cells. Accordingly, MSC-CM in NGF/p75 dependent manner induced apoptosis of HSCs and alleviated liver fibrosis [79].

TGF-β signaling is crucially important for pro-fibrotic function of HSCs [78]. Injection of MSC-CM decreased production of pro-fibrotic TGF-β in liver-infiltrated M1 macrophages which resulted in attenuated activation of HSCs and reduced fibrosis [14]. Additionally, MSC-CM treatment promoted generation of immunosuppressive, alternatively activated M2 macrophages which secreted anti-fibrotic molecules (C-C motif chemokine ligand 1 (CCL-1), IL-10) and reduced deposition of collagen-1 in liver parenchyma [14].

Several research groups indicated that MSC-Exos attenuated liver fibrosis by suppressing collagen production in HSCs [13,80,82]. Li and colleagues demonstrated that human UCD-MSC-Exos inhibited phosphorylation of Smad2 in HSCs, suppressed TGF-β/Smad2 signaling and attenuated synthesis of collagen type 1 and 3 [80]. Hyun and coworkers highlighted the importance of microRNA (miR)-125b-bearing MSC-Exos which suppressed pro-fibrotic function of HSCs by impeding activation of Hedgehog/Smoothened signaling pathway in these cells [82]. In line with these findings, Lou and associates suggested that miR-122 had a crucially important role in suppression of HSC-mediated liver fibrosis. By delivering miR-122 into HSCs, AT-MSC-Exos down-regulated expression of P4HA1 and IGF1R genes which controlled collagen production in HSCs [13].

### 4.2. MSC-Sourced Secretome in the Therapy of Lung Diseases

Beneficial effects of MSC-CM and MSC-Exo-based therapy have been demonstrated in the animal models of ALI, asthma, COPD and IPF (Table 2) [19,20,21,22,83,84,85,86,87,88,89,90,91]. Importantly, MSC-CM and MSC-EVs managed to induce regeneration of injured epithelium, attenuation of inflammation and fibrosis in the lungs in similar manner as transplanted MSCs [19,24,84,85,86,87].

Plenty of evidence suggest that MSC-derived secretome may be used in cell-free therapy of ALI [19,20,21,22,83]. Intratracheal administration of human BM-MSC-EVs enhanced repair and regeneration of injured alveolar epithelium in keratinocyte growth factor (KGF)-dependent manner [19]. MSC-derived KGF prevents or attenuates oxidant-mediated lung injury by increasing DNA repair capacity in pulmonary epithelial cells and by promoting alveolar fluid clearance through up-regulation of α1 subunit of Na^+^-K^+^-ATPase in alveolar type II epithelial cells [20,21]. In line with these findings are results obtained by Monsel and co-workers who demonstrated that human BM-MSC-EVs significantly attenuated ALI by enhancing bacterial clearance in KGF-dependent manner [22]. Moreover, human BM-MSC-Exos were able to induce generation of immunosuppressive phenotype in alveolar macrophages [83]. Intranasal administration of BM-MSC-Exo-primed alveolar macrophages created immunosuppressive microenvironment in injured lungs and attenuated endotoxin-induced ALI [83].

**Figure 1:**
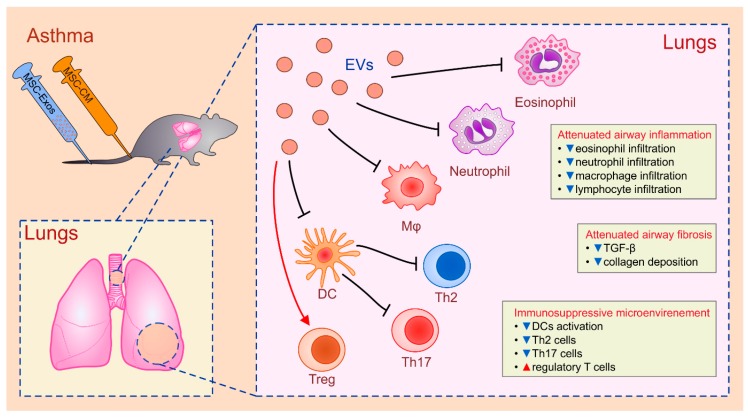
Molecular mechanisms responsible for beneficial effects of MSC-derived secretome in asthma. Administration of MSC-sourced secretome significantly reduced influx of circulating eosinophils, neutrophils, monocytes and lymphocytes in asthmatic lungs resulting in alleviation of on-going inflammation. MSC-CM or MSC-Exos reduced TGF-β production, decreased collagen deposition and attenuated fibrosis in the lungs. Additionally, MSC-derived secretome attenuated antigen-presenting function of DCs and suppressed Th2 and Th17 cell-driven inflammatory response in asthmatic lungs, but increased total number of lung-infiltrated IL-10-producing Tregs which created immunosuppressive microenvironment that allowed better functional recovery of asthmatic animals.

Several research groups [84,85,86] demonstrated that administration of MSC-CM and MSC-Exos alleviated inflammation and airway remodeling in asthmatic animals (Figure 1). De Castro and co-workers demonstrated that human AT-MSCs-Exos significantly attenuated ovalbumin (OVA)-induced allergic asthma in immunocompetent mice. AT-MSCs-Exos down-regulated total number of lung-infiltrated eosinophils and reduced expression of pro-fibrotic TGF-β in asthmatic lungs which resulted in decreased collagen fiber deposition [85]. Cruz and colleagues showed that systemic injection of BM-MSC-CM or BM-MSC-Exos significantly attenuated influx or inflammatory neutrophils, eosinophils, lymphocytes and macrophages in asthmatic murine lungs [84]. Importantly, BM-MSC-CM and BM-MSC-Exos were more potent than BM-MSCs in reducing the total number of neutrophils and eosinophils in the lungs. Additionally, BM-MSC-sourced secretome altered phenotype and function of lung-infiltrated antigen-specific CD4+T cells, resulting in attenuated airway inflammation. Detrimental Th2 and Th17 cell-driven inflammatory response in asthmatic lungs were suppressed by BM-MSC-derived secretome as evidence by significantly reduced number of IL-4, IL-5, and IL-17-producing CD4+T cells. Additionally, systemic administration of BM-MSC-sourced secretome increased total number of lung-infiltrated IL-10-producing CD4+ T cells and created immunosuppressive microenvironment in the lungs that allowed for better functional recovery of asthmatic animals [84]. Similar conclusions were made by Du and colleagues who confirmed in clinical settings that MSCs-Exos were able to successfully alleviate airway inflammation in asthmatic patients by modulating expansion and effector function of CD4+T cells [86]. MSC-Exos significantly attenuate antigen-presenting function of DCs and reduced their capacity for activation of naïve CD4+ T cells. Additionally, MSC-Exos promoted production of anti-inflammatory IL-10 and TGF-β in PB-MNCs of asthmatic patients and enhanced proliferative and immunosuppressive properties of Tregs [86].

Several lines of evidence suggested that beneficial effects of MSC-CM in COPD were a consequence of MSC-CM-induced inhibition of alveolar cell apoptosis or MSC-CM-based suppression of T cell:macrophage crosstalk in the lungs [23]. Administration of BM-MSC-CM reversed cigarette smoke-induced changes in caspase-3, p53, p21, p27, Akt, and p-Akt expression, suppressed collagen deposition and restored repair function of fibroblasts in the rat lungs [24]. MSC-CM-induced repair of injured alveolar epithelial cells was mainly relied on regenerative capacity of MSC-derived FGF-2 [23]. In line with these findings, Kim and colleagues designed FGF-2-bearing AT-MSC-sourced artificial nanovesicles which managed to efficiently alleviate COPD in mice by inducing proliferation of alveolar epithelial cells [25]. Importantly, lower doses of AT-MSC-derived artificial nanovesicles had beneficial effects similar to higher doses of AT-MSC-derived natural Exos, indicating that FGF-2-bearing artificial nanovesicles should be further explored in MSC-based cell-free therapy of COPD [25].

Although MSCs can be used for the attenuation of chronic lung inflammation and fibrosis, plenty of evidence suggest that aberrant activation of Wnt/β-catenin and TGF-β signaling pathways in lung resident MSCs may induce their differentiation in miofibroblasts and could, consequently, contribute to the development of IPF [87]. Having in mind that beneficial effects of MSCs in the therapy of IPF were mainly relied on their paracrine effects [88], Shantu and colleagues investigated whether MSC-sourced secretome may attenuate IPF as efficiently as MSCs [89]. They demonstrated that human BM-MSC-derived EVs managed to attenuate IPF by suppressing TGF-β-induced myofibroblastic differentiation of lung fibroblasts [89]. Thy-1-integrin interaction-dependent pathway was crucially important for the delivery of MSC-EVs components in the fibroblasts. Human BM-MSC-EVs are enriched with miRNAs with anti-fibrotic and immunomodulatory properties, including miR-199a/b-3p, 21-5p, 630, 22-3p, 196a-5p, 199b-5p, 34a-5p and 148a-3p [90]. Among them, miR-630, was mainly responsible for suppression of pro-fibrotic genes in lung fibroblasts. Administration of miR-630-containing MSC-EVs significantly reduced α-smooth muscle actin expression in lung fibroblasts and contributed to the MSC-EV-mediated alleviation of IPF [89,91].

### 4.3. Therapeutic Potential of MSC-Derived Secretome in Cartilage Regeneration

**Figure 2:**
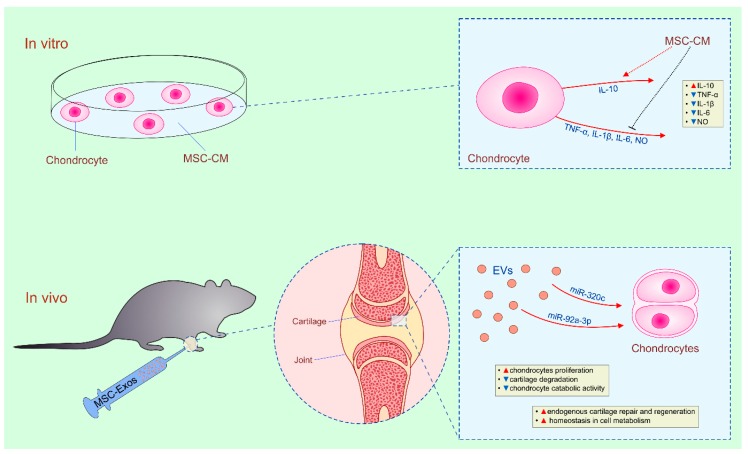
Herapeutic effects of MSC-derived secretome in cartilage regeneration. Chondrocytes cultured in the presence of MSC-derived secretome significantly reduce production of inflammatory cytokines which play detrimental role in cartilage degeneration during OA development (TNF-α, IL-1β, IL-6, nitric oxide (NO)) and increase production of immunosuppressive IL-10 which protects cartilage from inflammation-related injury. Accelerated neotissue filling and increased synthesis of type II collagen were noticed in osteoarthritic animals that received MSC-sourced secretome. MSC-derived extracellular vesicles (EVs) promoted endogenous cartilage repair and regeneration by delivering miR-320c and miR-92a-3p which restore homeostasis in bioenergetics and cell metabolism in proliferating chondrocytes.

Several lines of evidence suggested that beneficial effects of MSC-based therapy of osteoarthritis (OA) are, at least partially, mediated by MSC-sourced secretome (Table 3) [26,27,92,93,94,95,96]. Chen and colleagues showed that MSC-CM-treated chondrocytes significantly reduced production of inflammatory cytokines (TNF-α, IL-1β, IL-6), which play detrimental role in cartilage degeneration during OA development and progression (Figure 2) [92]. These findings are in line with results reported by Tofino-Vian and co-workers who demonstrated that IL-1β-activated OA chondrocytes were not capable to optimally produce inflammatory mediators (TNF-α, IL-1β, IL-6 and nitric oxide (NO)) in the presence of human AT-MSC-CM or AT-MSC-Exos [93]. Even more, AT-MSC-CM and AT-MSC-Exo-treatment enhanced production of immunosuppressive IL-10 in IL-1β-activated OA chondrocytes, indicating anti-inflammatory and chondroprotective effects of AT-MSC-derived secretome [93].

Therapeutic potential of MSC-sourced secretome was confirmed in vivo, as well. By using an immunocompetent rat osteochondral defect model, Zhang and colleagues demonstrated that multiple intra-articular injections (one/week for 12 weeks) of human MSC-Exos promoted cartilage repair and regeneration [26]. Accelerated neotissue filling and increased synthesis of type II collagen were noticed in OA lesions. MSC-Exos-treated OA rats displayed complete restoration of cartilage and subchondral bone with characteristic features including diffuse hypercellularity, good surface regularity and less bone erosions [26]. By analyzing signaling pathways involved in chondrocyte growth and proliferation, Toh and colleagues proposed that intra-articular administration of MSC-Exos promoted endogenous cartilage repair and regeneration by restoring homeostasis in bioenergetics and cell metabolism in proliferating chondrocytes. MSC-Exos contain glycolytic enzymes phosphoglucokinase and pyruvate kinase which activity restores redox potential in chondrocytes and facilitates regeneration of OA cartilage [27]. In addition, by delivering ATP-generating enzymes (adenylate kinase and nucleoside-diphosphate kinase), MSC-Exos may compensate reduced mitochondrial ATP production in OA chondrocytes enabling their enhanced proliferation. MSC-Exos express CD73, ecto 5-nucleotidase which degrades AMP to adenosine which, in turn, phosphorylates and activates survival kinases (Erk1/2 and Akt) [97]. Thus, when newly generated ATP was hydrolyzed to AMP, MSC-Exos, in CD73-dependent manner, converted AMP to adenosine and generated Erk1/2 and Akt-driven pro-survival signal in chondrocytes that initiated their proliferation resulting in enhanced regeneration of OA cartilage [27].

Keeping in mind that MSC-Exos contain miRNAs that regulate Erk and Akt pathways, several research groups investigated the role of miRNAs for MSC-Exo-dependent cartilage regeneration [98,99,100]. Sun and co-workers observed that human BM-MSCs, during differentiation into chondrocytes, up-regulated expression of 35 exosomal miRNAs (including miR-1246, miR-1290, miR-193a-5p, miR-320c, and miR-92a) and down-regulated expression of 106 miRNAs (including miR-377-3p and miR-6891-5p) [98]. Transfer experiments revealed that, among different miRNAs, miR-320c and miR-92a-3p-bearing BM-MSC-Exos the most efficiently promoted chondrogenesis in OA animals [98,99]. MSC-Exo-dependent delivery of miR-320c in chondrocytes promoted their proliferation, while miR-92a-3p reduced cartilage degradation by targeting Wnt5A protein which was responsible for increased chondrocyte catabolic activity in OA cartilage [96,98,99].

In an analogy, Liu and colleagues recently demonstrated that therapeutic effects of MSC-Exos in cartilage regeneration were particularly related to the activity of lncRNA KLF3 Antisense RNA 1 (KLF3-AS1), which acted as a competitive endogenous RNA that segregated miRNA206 away from its target G-protein-coupled receptor kinase interacting protein 1 (GIT-1) [94,95]. GIT-1 and miRNA206 have opposite effects on chondrogenesis. While GIT-1 prevents apoptosis of chondrocytes and, therefore, promotes cartilage regeneration and chondrogenesis [100], miRNA206 inhibits proliferation of chondrocytes and enhances cartilage degradation [100]. Accordingly, upon intra-articular administration, KLF3-AS1-bearing MSC-Exos were taken up by proliferating chondrocytes in injured cartilage of OA animals and, by suppressing miRNA206-based inhibition of GIT-1 activity, prevented apoptosis and enhanced proliferative capacity of chondrocytes resulting in cartilage repair and regeneration [95]. Therefore, significantly enhanced expression of cartilage specific gene Col2A1 (which encodes the alpha-1 chain of type II collagen) and cartilage specific protein aggrecan accompanied with down-regulated expression of cartilage degrading matrix metalloproteinase (MMP)-13 resulted in increased cartilage thickness that was observed in KLF3-AS1-MSC-Exo-treated OA rats [95].

### 4.4. Attenuation of Inflammatory Bowel Diseases by MSC-Derived Secretome

MSCs may suppress detrimental immune response in the gut, and were, therefore, used in cell-based therapy of inflammatory bowel diseases (IBDs). However,, results obtained in several clinical studies indicated that transplanted MSCs may either attenuate or aggravate colon inflammation [101,102,103]. It was concluded that engrafted MSCs polarized either in pro-inflammatory or anti-inflammatory cells in dependence of the concentration of inflammatory cytokines in the injured gut. After engraftment in the gut of patients with dominant Th1 or Th17 immune response (manifested by elevated concentration of interferon gamma (IFN-γ), tumor necrosis factor alpha (TNF-α), and interleukin (IL)-17) MSCs developed an anti-inflammatory phenotype, produced immunosuppressive IL-10 and KYN which efficiently inhibit proliferation, activation and effector function of inflammatory M1 macrophages, Th1 and Th17 cells, and alleviated CD. On contrary, after engraftment in the gut with low levels of Th1/Th17 inflammatory cytokines, MSCs adopted a pro-inflammatory phenotype, produced large amounts of inflammatory mediators which promoted migration and activation of neutrophils and effector T cells resulting in aggravation of CD [101,102,103].

**Figure 3:**
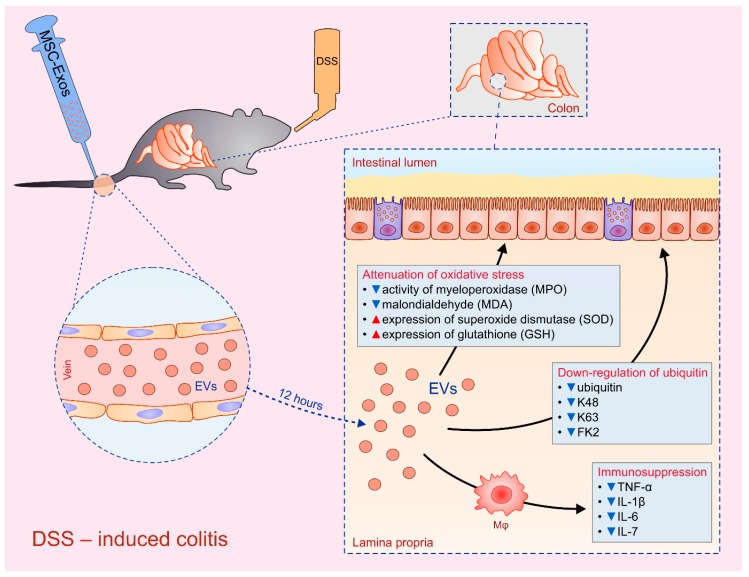
Therapeutic effects of MSC-derived secretome in attenuation of experimental colitis. Administration of MSC-sourced extracellular vesicles (EVs), including MSC-derived exosomes (Exos), efficiently alleviated dextran sodium sulphate (DSS)-induced colitis. Intravenous injection of MSC-EVs significantly decreased activity of myeloperoxidase (MPO), malondialdehyde (MDA) and notably increased expression of superoxide dismutase (SOD) and glutathione (GSH) in inflamed colons, indicating that modulation of anti-oxidant/oxidant balance in inflamed gut had important role for MSC-EVs-based therapeutic effects. Down-regulation of ubiquitin and ubiquitin-associated molecules (K48, K63 and FK2) in inflamed gut were also responsible for MSC-Exo-based attenuation of colitis. Additionally, MSC-derived secretome attenuated production of inflammatory cytokines (TNF-α, IL-1β, IL-6, IL-7) in colon macrophages resulting in alleviation of on-going inflammation.

In order to avoid unwanted effects of MSC-based therapy and, at the same time, utilize their immunosuppressive potential, several research groups [28,104,105,106] investigated therapeutic potential of MSC-sourced secretome as MSC-based, cell-free therapy for IBD (Table 4). Mao and colleagues demonstrated beneficial effects of human UCD-MSC-Exos in alleviation of dextran sodium sulphate (DSS)-induced colitis (Figure 3). MSC-Exos were detected in inflamed colons 12 hours after intravenous administration where, mainly by suppressing production of inflammatory cytokines in colon-infiltrating macrophages, attenuated on-going inflammation [28]. Among inflammatory cytokines, UCD-MSC-Exo-based therapy particularly down-regulated expression of IL-7 which promoted mucosal inflammation in the gut by acting as a mitogen and survival factor for T cells [28,105,106].

In line with these findings, Yang and co-workers demonstrated that inhibition of NF-kB p65-signaling pathway in colon-infiltrated immune cells, attenuation of oxidative stress and inhibition of apoptosis were mainly responsible for beneficial effects of MSC-derived secretome in IBD therapy [29]. Significantly decreased activity of myeloperoxidase (MPO), malondialdehyde (MDA) and notably increased expression of superoxide dismutase (SOD) and glutathione (GSH) in injured colons of BM-MSC-EVs-treated animals, indicating that modulation of anti-oxidant/oxidant balance in inflamed gut had important role for BM-MSC-EVs-based therapeutic effects. Additionally, significantly reduced cleavage of caspase-3,-8 and -9, observed in injured colons of BM-MSC-EVs-treated animals, suggested that modulation of apoptosis was also, at least partially, responsible for beneficial effects of BM-MSC-sourced secretome [29].

Ubiquitin is up-regulated in colitis and its down-regulation inhibits on-going inflammation in the gastrointestinal tract [30]. In line with these findings, Wu and colleagues suggested that down-regulation of ubiquitin in inflamed gut could be responsible for MSC-Exo-based attenuation of colitis since expression of ubiquitin and ubiquitin-associated molecules (K48, K63 and FK2) were significantly decreased in DSS-treated mice after injection of UCD-MSC-Exos [107].

### 4.5. MSC-Sourced Secretome as an Emerging Tool for Myocardial Regeneration

A large number of experimental studies demonstrated that MSC-derived secretome, particularly MSC-Exos, may be used for cardiac regeneration (Table 5) [31,32,33,108,109,110,111,112,113,114,115,116,117,118,119,120,121] Importantly, cardioprotective effects of MSC-EVs corresponded to beneficial effects of their parental MSCs [31,32,33]. By using an animal model of myocardial ischemia/reperfusion injury, Lai and colleagues showed that MSC-Exos significantly reduced infarct size [31] while Arslan and co-workers revealed that MSC-Exos enhanced myocardial viability through the activation of PI3K/Akt signaling pathway [32]. Cardioprotective role of MSC-Exos was also demonstrated by Yu and colleagues who observed significantly improved contractility of cardiomyocytes and notably reduced infarct size in MSC-Exo-treated rats [33].

**Figure 4:**
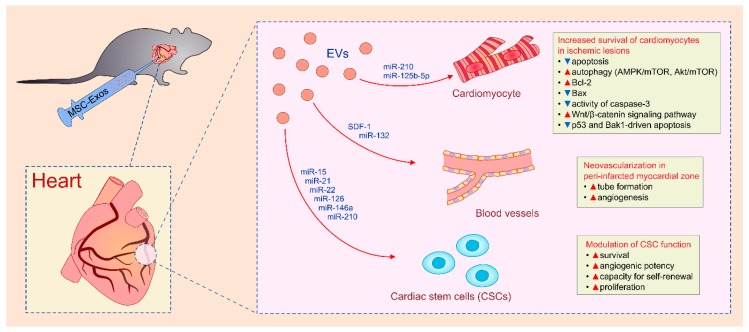
Therapeutic potential of MSC-sourced secretome in myocardial regeneration. MSC-derived secretome promote myocardial regeneration by preventing apoptosis of cardiomyocytes, by inducing neo-angiogenesis in ischemic regions and by promoting survival, angiogenic potency and capacity for self-renewal of cardiac stem cells (CSCs). MSC-derived extracellular vesicles (EVs) increased survival of cardiomyocytes in ischemic lesions by preventing apoptosis and by inducing autophagy via modulation of AMPK/mTOR, Akt/mTOR and Wnt/β-catenin pathways. Administration of MSC-derived exosomes (Exos) resulted in up-regulation of anti-apoptotic Bcl-2, down-regulation of pro-apoptotic Bax and suppressed activity of caspase-3 in cardiomyocytes. MSC-Exos-mediated delivery of miR-210 and miR-125b-5p increased survival of cardiomyocytes by preventing p53 and Bak1-driven apoptosis. MSC-Exo-mediated delivery of stromal cell-derived factor-1 (SDF-1) and miR-132 resulted in enhanced tube formation and increased angiogenic capacity of endothelial cells (ECs). MSC-Exos-mediated modulation of CSCs function has been attributed to the delivery of miR-15, miR-21, miR-22, miR-126, miR-146a, miR-210 which prevented apoptosis and promoted survival of CSCs.

Cardiomyocytes, ECs and cardiac stem cells (CSCs) were the main cellular targets in MSC-Exos-based cardiac regeneration (Figure 4) [109,110]. Intramyocardial injection of MSC-Exos induced cardiomyocyte proliferation and neo-angiogenesis which were manifested by significantly improved cardiac function and increased capillary density in ischemic zones of infarcted hearts [109]. MSC-Exos rescued myocardial ischaemia/reperfusion injury and reduced myocardial infarct size in experimental animals. Mechanistically, MSC-Exos increased survival of cardiomyocytes in ischemic lesions by preventing apoptosis and by inducing autophagy via AMPK/mTOR and Akt/mTOR pathways [111]. Administration of MSC-Exos resulted in up-regulation of anti-apoptotic Bcl-2, down-regulation of pro-apoptotic Bax and suppressed activity of caspase-3 in cardiomyocytes [112]. Cui and coworkers showed that MSC-Exos protected cardiomyocytes against apoptosis through the activation of Wnt/β-catenin signaling pathway since pharmacological inhibition of this cascade neutralized MSC-Exos-induced anti-apoptotic and cardioprotective effects [112]. Additionally, as recently demonstrated by Zhu and colleagues, MSC-Exos delivered miR-210 and miR-125b-5p in cardiomyocytes, and increased their survival by preventing p53 and Bak1-driven apoptosis [113,114]. Importantly, hypoxia significantly enriched miR-210 and miR-125b-5p content in MSC-Exos and enhanced their cardioprotective effects [113,114]. Significantly higher survival, smaller scar size and better cardiac function were observed in animals that received MSC-Exos obtained from MSCs which were cultured in hypoxic conditions compared to those that received Exos derived from MSCs that grew under standard culture conditions [114]. The positive effect of hypoxia on MSC-Exo-based cardioprotection was relied on expression of neutral sphingomyelinase 2 (nSMase2) which regulated miR-210 secretion [114]. Inhibition of nSMase2 activity significantly reduced miR-210 secretion and completely abrogated beneficial effects of MSC-Exos in myocardial repair [114].

Modulation of angiogenesis in peri-infarcted myocardial zone was, at least partially, responsible for MSC-Exo-induced beneficial effects [115]. In line with our recently published study that emphasized the important role of stromal cell-derived factor 1 (SDF-1) in neo-angiogenesis [116], Gong and colleagues showed that SDF-1-overexpression in MSCs-Exos inhibited apoptosis of cardiomyocytes by promoting generation of new blood vessels in peri-infarcted myocardial zone [117]. As demonstrated by Ma and colleagues [109], miR-132 was also involved in MSC-Exo-induced neovascularization in ischemic hearts. MSC-Exo-mediated delivery of miR-132 resulted in enhanced tube formation and increased angiogenic capacity of ECs [118].

Administration of MSC-Exos significantly improved stem cell properties and regenerative potential of CSCs [119]. Survival, angiogenic potency and capacity for self-renewal were significantly improved in MSC-Exo-primed CSCs [119]. MSC-Exo-mediated modulation of CSC function has been attributed to the delivery of specific microRNAs (miR-15, miR-21, miR-22, miR-126, miR-146a, miR-210) which prevented apoptosis and promoted survival and proliferation of CSCs [110,119,120].

In addition to alleviation of myocardial ischemia/reperfusion injury, MSC-Exos efficiently attenuated dilated cardiomyopathy [121]. Significantly improved myocardial function, attenuated cardiac dilation and reduced apoptosis of cardiomyocytes were observed in animals that intravenously received MSC-Exos. Beneficial effects of MSC-Exos were mainly relied on their anti-inflammatory effects. MSC-Exos improved the inflammatory microenvironment in the hearts by regulating function of macrophages, which were crucially important for the development of myocardial inflammation in dilated cardiomyopathy. Systemic injection of MSC-Exos remarkably attenuated the total number of pro-inflammatory macrophages in the hearts and significantly decreased serum concentration of macrophage-derived inflammatory cytokines and chemokines which reduced influx of circulating inflammatory cells in the MSC-Exos treated hearts [121].

### 4.6. Beneficial Effects of MSC-Derived Secretome in the Therapy of Eye Disease

Therapeutic potential of MSC-sourced secretome had been demonstrated in attenuation of several degenerative and inflammatory eye diseases including glaucoma, autoimmune uveitis, corneal injury, dry eye disease (DED) and Sly syndrome (Table 6) [34,35,43,122,123,124,125,126,127,128,129,130,131,132].

**Figure 5:**
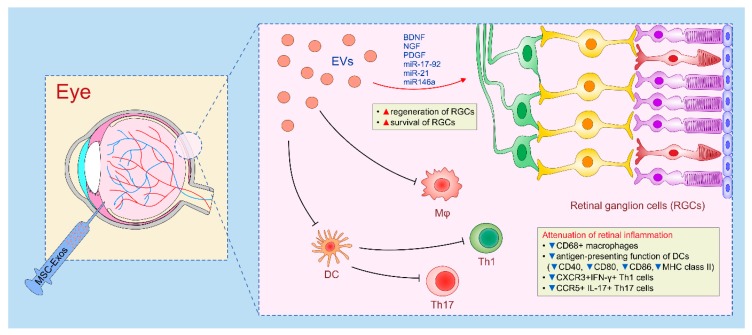
Molecular mechanisms responsible for beneficial effects of MSC-derived secretome in retinal regeneration. MSC-derived exosomes (Exos) promote regeneration of injured retina by supplying retinal ganglion cells (RGCs) with miR-17-92, miR-21, miR146a and neurotrophins (brain-derived neurotrophic factor (BDNF), nerve growth factor (NGF) and platelet-derived growth factor (PDGF)). MSC-sourced secretome suppress detrimental immune response in the eye through the inhibition of antigen-presenting cells (macrophages and dendritic cells (DCs)) which results in attenuated activation of Th1 and Th17 cells and alleviation of retinal injury and inflammation.

MSC-Exos are able to reside in the vitreous humor at least one month after intravitreal administration and, due to their nano dimension, may rapidly reach retinal ganglion cells (RGCs) which are gradually lost during glaucoma progression [43]. As demonstrated by Mead and colleagues, BM-MSC-Exos diffused rapidly throughout the retina and, within one hour after their intravitreal injection, BM-MSC-Exos successfully delivered neurotrophins (BDNF, NGF and PDGF) to the injured RGCs promoting their survival and regeneration (Figure 5) [122,123,124]. Therefore, therapeutic effects of BM-MSC-Exos in glaucoma treatment were similar to those observed in BM-MSC-treated animals [43,122,123,124]. Importantly, these beneficial effects were not noticed after intravitreal injection of fibroblasts-derived Exos, indicating specific therapeutic potential of MSCs-Exos in RGCs regeneration and glaucoma treatment [124]. However, beneficial effects of BM-MSC-Exos were only observed when BM-MSC-Exos were continuously injected (at least once per week) in glaucomatous eyes while longer delays between treatments completely abrogated MSC-Exo-dependent effects [124].

Beneficial effects of MSC-Exos in glaucoma treatment were relied on activity of MSC-derived miRNAs [43]. Knockdown of Argonaute2 protein, which is crucially important for miRNA function, significantly attenuated BM-MSC-Exo-induced effects [124]. RNA sequencing revealed that more than 40 miRNAs were up-regulated in BM-MSC-Exos, compared to fibroblast-derived Exos, and, among them, miR-17-92, miR-21 and miR146a were designated as the most important for regeneration of RGCs in glaucomatous eyes [122,123]. Expression of phosphatase and tensin homolog (PTEN), which is an important suppressor of RGC axonal growth and survival, were regulated by miR-17-92 and miR-21 while miR-146a modulated expression of EGFR involved in inhibition of axon regeneration [43].

By using animal model of laser-induced retinal injury, Yu and co-workers demonstrated that MSC-Exos supply injured retinas with immunomodulatory factors which results in alleviation of retinal inflammation [34]. Attenuated laser-induced retinal injury, observed in MSC-Exo-treated eyes, was accompanied by an increased number of photoreceptor cells and significantly reduced number of inflammatory cells, particularly CD68+ macrophages. Cellular make-up of the retinas revealed that MSC-Exos suppressed MCP-1-dependent migration of monocytes in injured retinas and attenuated TNF-α-driven retinal inflammation. Expression of macrophage-derived TNF-α and MCP-1 were down-regulated in MSC-Exo-treated retinas. Application of MCP-1 completely diminished immunosuppressive and therapeutic effects of MSC-Exos and significantly aggravated macrophage-driven inflammation and laser induced injury [34].

MSC-Exos suppressed detrimental immune response in the eye during the attenuation of experimental autoimmune uveitis (EAU) and corneal injury [122]. As demonstrated by Bai and colleagues, periocular injection of MSC-Exos attenuated EAU by reducing MCP-1 and CCL21-dependent influx of neutrophils, NK cells, macrophages and T cells in inflamed retinas [35]. Among effector T cells, MSC-Exos selectively prevented influx of CXCR3-expressing, IFN-γ producing Th1 and CCR5-expressing IL-17 producing Th17 cells in inflamed retinas, without affecting migration of immunosuppressive T regs [35]. Similar to these results were findings obtained by Shigemoto-Kuroda and colleagues [125] who demonstrated that suppression of Th1 and Th17 immune response was a consequence of MSC-Exo-based attenuation of antigen-presenting function of DCs. Flow cytometry analysis of MSC-Exo-treated DCs revealed down-regulated expression of co-stimulatory molecules (CD40, CD80 and CD86) and reduced expression of MHC class II molecules [125]. Additionally, the transcript levels of Th1 (IL-12, IFN-γ) and Th17-related inflammatory cytokines (IL-1β, IL-6, and IL-17A) were significantly lower in the eyes of MSCs-Exos-treated mice when compared to the vehicle-treated controls, indicating that the main mechanism of MSC-Exos-mediated attenuation of EAU was relied on suppression of DC-driven generation of Th1 and Th17 immune response [35,125].

In a similar manner as in EAU, detrimental immune response has crucially important role in the pathogenesis of corneal injury and DED, multifactorial diseases of the ocular surface and tears that result in visual disturbances [122]. It is well known that IL-1β-producing macrophages orchestrate influx of circulating leukocytes in injured corneas while Th17 cell-derived IL-17A and IL-22 regulate progression of DED [126,127,128]. Accordingly, suppression of IL-1β-driven inflammation in corneal tissue and attenuation of Th17 immune response resulted in alleviation of corneal injury and DED [122]. MSC-derived IL-1Ra attenuate production of inflammatory cytokines (IL-1β and TNF-α) in M1 macrophages and promotes their polarization towards immunosuppressive, IL-10-producing M2 phenotype [129]. Similarly, DCs cultured in the presence of MSCs-derived IDO-1 and growth-related oncogene (GRO) developed tolerogenic and immunosuppressive phenotype and, instead of Th17-related inflammatory cytokines, produced large amounts of anti-inflammatory IL-10 [60,130]. In line with these observations, we recently developed an immunomodulatory ophthalmic solution (“Exosomes Derived Multiple Allogeneic Proteins Paracrine Signaling (Exosomes D-MAPPS)”) whose activity is based on the capacity of MSC-Exos to suppress immune response in IL-1Ra, GRO and IDO-1/KYN-dependent manner having beneficial effects in the treatment of corneal injuries an DED [43,131].

In addition to degenerative and inflammatory diseases, MSC-derived secretome efficiently alleviated Mucopolysaccharidosis VII (Sly Syndrome), corneal congenital metabolic disease caused by a mutation of β-glucuronidase, enzyme required for the degradation of glycosaminoglycans (GAGs) [132]. Coulson-Thomas and colleagues demonstrated that, after intraocular administration, UCD-MSC-Exos delivered β-glucuronidase into the keratocytes and enabled degradation of accumulated GAGs. These findings indicate that UCD-MSC-Exos should be further explored as new, cell-free vehicles for enzyme substitution therapy of inherited metabolic diseases.

### 4.7. MSC-Derived Secretomes in the Therapy of Ischemic Brain Damage and Spinal Cord Injury 

Several lines of evidence [37,133,134,135,136,137,138,139,140] suggested that MSC-sourced secretome may be used in neural regeneration (Table 7). By using a rat model of ischemic brain injury, Xin and colleagues demonstrated that intravenous transplantation of MSC-Exos significantly improved neurogenesis and neurite remodeling [133]. Histological analysis revealed that MSC-Exos-based therapy promoted axonal growth and significantly increased presence of neuroblasts and ECs in ischemic regions. Four weeks after injury, axonal density was significantly increased along the ischemic boundary zone of the cortex and striatum in MSC-Exo-treated rats [133]. MSCs-Exo regulated neurogenesis by supplying neurons with miR-124 and miR-133b which promotes neurite outgrowth by targeting Ras homolog gene family member A (RhoA) [37].

**Figure 6:**
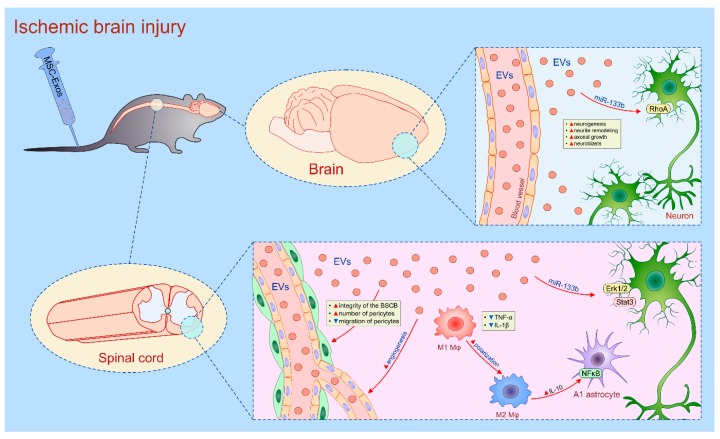
Molecular mechanisms responsible for beneficial effects of MSC-derived secretomes in the therapy of ischemic brain damage and spinal cord injury. Administration of MSC-sourced extracellular vesicles (EVs), including MSC-derived exosomes (Exos), promoted neural regeneration in animal models of ischemic brain damage and spinal cord injury (SCI). MSC-Exos-based therapy improved neurogenesis, promoted axonal growth, increased presence of neuroblasts and endothelial cells (ECs) in ischemic regions of the brain. MSCs-Exo regulated neurogenesis by supplying neurons with miR-133b which promoted neurite outgrowth by targeting Ras homolog gene family member A (RhoA). Similarly, systemic injection of miR-133b-bearing MSC-Exos promoted recovery from SCI by promoting regeneration of axons through the activation of survival Erk1/2 and Stat-3 signaling pathways in regenerating neurons. After intravenous administration, MSC-Exos accumulated at the site of SCI and promoted generation of immunosuppressive M2 macrophages which, in IL-10-dependent manner, suppressed activation of neurotoxic A1 astrocytes through the inhibition of NF-kB. In similar manner, via down-regulation of NF-κB p65 signaling, MSC-EVs reduced migratory capacities of pericytes and maintained structural integrity of blood-spinal cord barrier (BSCB).

Similarly, Dong and co-workers recently demonstrated that systemic injection of miR-133b-bearing MSC-Exos promoted recovery from spinal cord injury (SCI) by promoting regeneration of axons through the activation of survival Erk1/2 and Stat-3 signaling pathways in neurons. Importantly, miR-133b-bearing-MSC-Exos significantly improved recovery of hindlimb locomotor function in experimental rats [134], indicating that these MSC-EVs should be further explored as new, cell-free therapeutic agents for the treatment of SCI (Figure 6).

In addition to their direct neuroprotective effects of injured neurons, MSC-Exos are also able to modulate microenvironment of spinal cord lesions through their anti-inflammatory and pro-angiogenic effects [135,136]. As evidenced by Huang and colleagues, systemic application of MSC-Exos promoted functional recovery following SCI by inducing neo-angiogenesis and through the suppression of TNF-α and IL-1β-driven inflammation [135]. Furthermore, MSC-Exo treatment enhanced production of immunosuppressive IL-10 which suppressed neurotoxic A1 astrocytes [135]. In line with these findings, Wang and co-workers showed that MSC-Exos may prevent pro-inflammatory properties of A1 astrocytes by inhibiting nuclear translocation of p65 subunit of NF-κB, which is crucially important for the generation of inflammatory phenotype in these cells [137]. In addition to N1 astrocytes, macrophages were the main cellular targets in MSC-Exo-based immunomodulation of SCI [136,138]. After intravenous administration, MSC-Exos accumulated at the site of SCI where promoted polarization of inflammatory M1 macrophages into immunosuppressive M2 phenotype. Accordingly, enhanced presence of CD206-expressing and IL-10-producing M2 macrophages and reduced number of TNF-α and IL-1β-producing M1 macrophages were observed in spinal cord lesions of MSC-Exo-treated animals [136,138]. Having in mind that TNF-α and IL-1β-driven inflammation results in severe neuropathic pain, Shiue and colleagues investigated therapeutic potential of MSC-Exos in attenuation of nerve-injury induced pain [139]. They demonstrated that continuous intrathecal infusion of human UCD-MSC-Exos achieved excellent preventive and reversal effects for nerve ligation-induced pain. Analgesic effects of MSC-Exos were relied on the delivery of neurotrophins (BDNF, glial cell line-derived neurotrophic factor (GDNF)) and immunosuppressive factors (IL-10) in the neurons and glial cells [139].

As recently revealed by Lu and colleagues, systemic administration of BM-MSC-EVs improved motor function in SCI-treated animals by preventing disruption of the blood-spinal cord barrier (BSCB) [140]. Since pericytes play a pivotal role in maintaining the structural integrity of BSCB, BM-MSC-EVs increased total number of pericytes in BSCB by reducing their migratory capacities via down-regulation of NF-κB p65 signaling [140].

## 5. Clinical Studies Addressing Therapeutic Potential of MSC-Derived Secretome

Although results obtained in animal models suggested beneficial effects of MSC-sourced secretome, only several clinical studies confirmed regenerative and immunomodulatory potential of MSC-CM and MSC-derived EVs. Administration of MSC-derived secretome efficiently improved clinical outcomes in patients suffering from severe alveolar bone atrophy, alopecia and graft-versus-host disease (GvHD) [141,142,143,144]. Importantly, adverse effects have not been reported in patients that received MSC-sourced secretome, indicating that local and systemic injection of MSC-CM and MSC-Exos is safe therapeutic approach [141,142,143,144].

In the case of alveolar bone regeneration, eight patients received either porous pure beta-tricalcium phosphate or shell-shaped atelocollagen sponge scaffold grafts soaked in the BM-MSC-CM [141]. Radiographic and histological evaluation revealed mineralization, early bone formation and reduced infiltration of inflammatory cells in patients that received BM-MSC-CM-containing scaffold grafts. Among MSC-derived immunomodulatory and trophic factors, VEGF, TGF-β, and HGF contributed to the beneficial effects of BM-MSC-secretome in bone regeneration [141].

Results obtained in clinical trials addressing alopecia [142] and Female Pattern Hair Loss (FPHL) [143] revealed that AT-MSC-CM may represent a new therapy for hair regeneration. Significantly increased hair density was observed in 22 patients with alopecia (11 men and 11 women) and in 27 patients with FPHL that intradermal received AT-MSC-CM (0.02 mL/cm^2^). Approximately a total volume of 3 to 4 mL of AT-MSC-CM was administered during each session of treatment. Patients received intradermal treatment of AT-MSC-CM every 3 to 5 weeks for a total of 6 sessions. Among MSC-derived growth factors, elevated levels of HGF, FGF-1, IL-6, VEGF and TGF-β were measured in AT-MSC-CM. Importantly, AT-MSC-CM was well tolerated and no side effects were observed in 49 patients that received multiple intradermal injections of AT-MSC-derived secretome [142,143].

MSC-Exos significantly improved symptoms of GvHD in a patient who suffered from treatment-refractory GvHD [144]. According to the application regime of MSCs in GvHD patients (0.4–9.0 × 10^6^ MSCs/kg body weight) [145] the amount of MSC-Exos obtained from the supernatant of 4 × 10^7^ MSCs was used as a 1 therapeutic unit [144]. To reduce the risk of potential side effects, only a tenth of an MSC-Exo unit was initially administered. Since no side effects were observed, unit amounts were gradually increased and 4 therapeutic units were administered every 2–3 days in next several months. Systemic injection of MSC-Exos was well tolerated and no side effects were observed during 7-month follow-up period. Among MSC-derived immunosuppressive factors, IL-10 and TGF-β were noticed in the highest concentrations in MSC-Exos. Accordingly, MSC-Exos impaired capability of patient’s PBMNCs to produce inflammatory cytokines (IL-1β, IFN-γ and TNF-α) and attenuated on-going inflammation in gut and skin [144]. Reduction of diarrhea volume and attenuation of cutaneous and mucosal symptoms associated with GvHD were observed two weeks after initial administration of MSC-Exos and were stable in next 4 months, indicating a long-lasting therapeutic effect of MSC-Exos [144].

In line with these findings, researchers from the Isfahan University of Medical Sciences decided to elucidate safety and efficacy of MSC-Exos on disability of patients with acute ischemic stroke. This clinical trial has not been started yet. Patients will, one month after ischemic injury, receive allogenic miR-124-expressing MSC-Exos via stereotaxis. Incidence of treatment-emergent side effects (stroke recurrences, brain edema, seizures) and improvement of disability will be monitored during this study (NCT03384433).

Similarly, researchers from the Punta Pacifica Hospital of Panama City decided to elucidate safety and efficacy of allogeneic UCD-MSC-derived trophic factors (MTF) in adult asthmatic patients. This study is still recruiting patients who will intra-nasally receive MTF once per week for a period of 4 weeks. Side effects as well as alterations of lung function will be monitored during one month follow up (NCT02192736).

## 6. Conclusions and Future Perspectives

**Figure 7:**
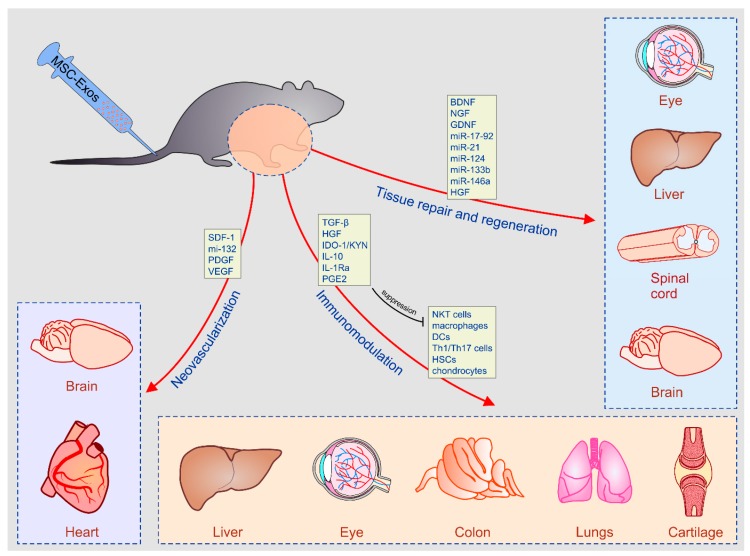
Molecular mechanisms responsible for beneficial effects of MSC-derived secretome in tissue repair and regeneration. Results obtained in experimental studies suggest that MSC-derived secretome represents a promising therapeutic tool for the treatment of degenerative and inflammatory diseases. Beneficial effects of MSC-sourced secretomes rely on their capacity to deliver neurotrophins (brain-derived neurotrophic factor (BDNF), nerve growth factor (NGF), hepatocyte growth factor (HGF), miR-17-92, miR-21, miR-124, miR-133b, miR146a which enable regeneration of injured liver, brain, spinal cord and eye. MSC-derived secretomes contain immunomodulatory factors which inhibit proliferation and activation of inflammatory immune cells and promote expansion of immunosuppressive cells resulting in alleviation of inflammation-related tissue injury. MSC-sourced secretomes are enriched with angiomodulatory factors (stromal cell derived factor-1 (SDF-1), miR-132, platelet-derived growth factor (PDGF), vascular endothelial growth factor (VEGF)) that promote angiogenesis and neo-vascularization in ischemic regions of brain and heart enhancing survival of injured neurons and cardiomyocytes.

Results obtained in experimental and clinical studies suggest that MSC-derived secretome represents a promising therapeutic tool for the treatment of degenerative and inflammatory diseases. Importantly, administration of MSC-CM and MSC-EVs were as effective as transplantation of the corresponding MSCs in attenuation of acute and chronic inflammatory diseases of gastrointestinal, respiratory, cardiovascular and central nervous system [19,20,21,22,28,29,31,32,33,37,72,74,97,98,99,122,123,124,127,137]. Beneficial effects of MSC-sourced secretomes rely on their capacity to deliver genetic material, growth and immunomodulatory factors to the target cells enabling activation of anti-apoptotic and pro-survival pathways which results in enhanced tissue repair and regeneration (Figure 7).

However, it should be noted that there are still several issues which limit potential clinical use of MSC-derived secretome. Therapeutic potential of MSC-sourced secretome depends on functional properties of MSCs from which it was obtained. Although MSCs have low expression of MHC molecules, several lines of evidence indicated that transplantation of allogeneic MSC can induce measurable allogeneic immune responses in MHC-mismatched recipients [146,147,148,149]. However, immunogenicity of MSC-sourced secretome is still a matter of debate. Lou and colleagues proposed that MSC-Exos are less immunogenic than their parent MSCs because of lower content in membrane-bound proteins including tetraspanins (CD81, CD63 and CD9), heat-shock proteins (HSP60, HSP70 and HSP90), programmed cell death 6-interacting protein and tumor susceptibility gene 101 [13]. In line with these observations are findings recently reported by Kordelas and coworkers who demonstrated that multiple injections of BM-MSC-Exos, obtained from four unrelated donors, did not evoke allogeneic immune response in MHC-mismatched recipient [144]. Nevertheless, evidence recently provided by Liu and coworkers indicated that potentially immunogenic proteins such as MHC molecules can also be transferred via EVs [150]. This raises the important safety concern for administration of MSC-EVs in MHC-mismatched recipients since MHC-bearing MSC-EVs could provoke detrimental allogeneic immune response. It should be emphasized that there is still no clear evidence that MSC-EVs are able to transfer MHC molecules to target cells that could result in generation of allogeneic immune response. Therefore, future experimental studies should be designed to investigate the influence of MHC-bearing MSC-EVs on immune response of MHC-mismatched recipients in order to delineate immunogenicity of MSC-derived secretome. 

Additionally, since sub-populations of MSCs differ in their capacity for differentiation and immunomodulation, heterogeneity of MSC-derived secretomes may cause diverse effects on their target cells. MSCs should be exposed to culture conditions which reflect a specific inflammatory microenvironment of the tissue that is going to be regenerated by MSC-derived secretome. Methods used to precondition MSCs in stimulating their functional properties, such as hypoxia and cytokine priming, significantly modify content and therapeutic effects of MSC-sourced secretome. Therefore, future experimental and clinical studies should precisely define protocols for generation of MSC-derived secretome for each of MSC subpopulations and for particular pathological conditions before MSC-sourced secretome will be offered worldwide as a universal human remedy.

## Figures and Tables

**Table 1:** Therapeutic potential of MSC-derived secretome in liver regeneration.

Source Cell Type	Type of Secretome	Target Cells/Condition	Effects/Major Findings	Pathways Involved	Ref. No.
BM-MSCs	Exos	leukocytes/ALF and liver fibrosis	inhibited activation of inflammasome	IDO-1/KYN; TGF-β; IL-10	[13,74]
AT-MSCs	Exos	HSCs/liver fibrosis	reduced collagen production	miR-122:Hedgehog/Smoothened	[13]
BM-MSCs	CM	macrophages/liver fibrosis	conversion from M1 to M2 phenotype	TGF-β/Smad	[14]
BM-MSC	CM	Th1 and Th17 cells/ALF	reduced influx in the injured liver	IL-10; CXCR3 and CCR5	[15,75]
BM-MSC	CM/Exos	hepatocytes/ALF	inhibition of apoptosis and enhanced proliferation	IDO-1/KYN; HGF; fibrinogen-like protein 1; IL-6/gp130; Bcl-xL; Cyclin D1	[16,17,74,76]
BM-MSC	CM	NKT cells/ALF	reduced production of inflammatory cytokines and attenuated cytotoxicity	IDO-1/KYN	[74,76]
BM-MSCs	CM	T cells/liver fibrosis	expansion of Tregs; suppression of Th17 cells	IDO-1/KYN	[18]
BM-MSCs	CM	HSCs/liver fibrosis	inhibited activation and enhanced apoptosis	IDO-1/KYN; IL-10; NGF/p75	[77,78]
UCD-MSCs	Exos	HSCs/liver fibrosis	reduced collagen production	TGF-β/Smad2	[80]

Abbreviations: bone marrow (BM); adipose tissue (AT); umbilical cord (UCD); mesenchymal stem cells (MSCs); conditioned medium (CM); exosomes (Exos); acute liver failure (ALF); interleukin (IL)-10; transforming growth factor beta (TGF-β); indoleamine 2,3 dioxygenase-1 (IDO-1); Kynurenine (KYN); hepatic stellate cells (HSCs); natural killer T cells (NKT); T regulatory cells (Tregs); C-X-C motif) receptor 3 (CXCR3); C-C chemokine receptor type 5 (CCR5); hepatocyte growth factor (HGF); nerve growth factor (NGF).

**Table 2:** MSC-sourced secretome in the therapy of lung diseases.

Source Cell Type	Type of Secretome	Target Cells/Condition	Effects/Major Findings	Pathways Involved	Ref. No.
BM-MSCs	EVs	alveolar type II epithelial cells/ALI	attenuation of oxidant-mediated injury	KGF	[19,20,21,22]
BM-MSCs	Exos	alveolar macrophages/ALI	generation of immunosuppressive phenotype	IL-10; TGF-β	[83]
AT-MSCs/BM-MSCs	CM/Exos	neutrophils; eosinophils; Th2 and Th17 cells/asthma	suppression of cytokine production	IL-10	[84,85]
BM-MSCs	Exos	DCs/asthma	attenuated antigen presenting function	IL-10; TGF-β	[86]
BM-MSCs/AT-MSCs	CM	alveolar type II epithelial cells; fibroblasts/COPD	attenuated apoptosis; suppressed collagen deposition	FGF-2	[23,24,25]
BM-MSCs	EVs	fibroblasts/IPF	suppressed myofibroblastic differentiation	miR-630	[89,91]

Abbreviations: bone marrow (BM); adipose tissue (AT); mesenchymal stem cells (MSCs); conditioned medium (CM); exosomes (Exos); extracellular vesicles (EVs); acute lung injury (ALI); keratinocyte growth factor (KGF); interleukin (IL)-10; transforming growth factor beta (TGF-β); dendritic cells (DCs); chronic obstructive pulmonary disease (COPD); fibroblast growth factor 2 (FGF-2); idiopathic pulmonary fibrosis (IPF).

**Table 3:** Therapeutic potential of MSC-derived secretome in cartilage regeneration.

Source Cell Type	Type of Secretome	Target Cells/Condition	Effects/Major Findings	Pathways Involved	Ref. No.
BM-MSCs/AT-MSCs	CM/Exos	chondrocytes/in vitro	reduced production of inflammatory cytokines	IL-10	[92,93]
BM-MSCs	Exos	chondrocytes/OA	restoration of homeostasis in bioenergetics and cell metabolism/restoration of cartilage and subchondral bone	adenylate kinase and nucleoside-diphosphate kinase-dependent pathways	[26,27]
BM-MSCs	Exos	chondrocytes/OA	enhanced proliferation/cartilage regeneration	miR-320c and miR-92a–3p	[98,99]
BM-MSCs	Exos	chondrocytes/OA	reduced apoptosis and enhanced proliferation/cartilage regeneration	KLF3-AS1	[100]

Abbreviations: bone marrow (BM); adipose tissue (AT); mesenchymal stem cells (MSCs); conditioned medium (CM); exosomes (Exos); osteoarthritis (OA); interleukin (IL)-10; microRNA (miR); lncRNA KLF3 Antisense RNA 1 (KLF3-AS1).

**Table 4:** Therapeutic potential of MSC-derived secretome in attenuation of inflammatory bowel diseases.

Source Cell Type	Type of Secretome	Target Cells/Condition	Effects/Major Findings	Pathways Involved	Ref. No.
UCD-MSCs	Exos	macrophages/colitis	reduced production of inflammatory cytokines	IL-10/IL-7	[28]
BM-MSCs	EVs	leukocytes/colitis	reduced production of inflammatory cytokines	NF-kB-p65	[29]
BM-MSCs	EVs	epithelial cell/colitis	attenuation of oxidative stress and inhibition of apoptosis	MPO, MDA, SOD, GSH/caspase-3,-8 and -9	[29]
BM-MSCs	Exos	epithelial cell/colitis	down-regulated expression of ubiquitin and ubiquitin-associated molecules (K48, K63 and FK2)	IL-10; IDO-1/KYN	[30]

Abbreviations: umbilical cord (UCD); bone marrow (BM); mesenchymal stem cells (MSCs); exosomes (Exos); extracellular vesicles (EVs); interleukin (IL)-10; myeloperoxidase (MPO), malondialdehyde (MDA); superoxide dismutase (SOD); glutathione (GSH); nuclear factor-kB (NF-kB); indoleamine 2,3 dioxygenase-1 (IDO-1); Kynurenine (KYN).

**Table 5:** Therapeutic potential of MSC-sourced secretome in myocardial regeneration.

Source Cell Type	Type of Secretome	Target Cells/Condition	Effects/Major Findings	Pathways Involved	Ref. No.
BM-MSCs	Exos	cardiomyocytes/myocardial ischemia/reperfusion injury	improved contractility/reduced infarct size	PI3K/Akt	[31,32,33]
BM-MSCs	Exos	cardiomyocytes/myocardial ischemia/reperfusion injury	prevention of apoptosis and induction of autophagy/reduced myocardial infarct size	AMPK/mTOR and Akt/mTOR	[111]
BM-MSCs	Exos	cardiomyocytes/myocardial ischemia/reperfusion injury	prevention of apoptosis and increased survival/reduced myocardial infarct size	Bcl-2, Bax, caspase-3; Wnt/β-catenin	[112]
BM-MSCs	Exos	cardiomyocytes/myocardial ischemia/reperfusion injury	increased survival/reduced infarct size	miR-210 and miR-125b-5p	[113,114]
BM-MSCs	Exos	cardiomyocytes/myocardial ischemia/reperfusion injury	higher survival/smaller scar size/better cardiac function	neutral sphingomyelinase /miR-210	[114]
BM-MSCs	Exos	endothelial cells/ischemia/reperfusion injury	generation of new blood vessels in peri-infarcted myocardial zone	SDF-1/miR-132	[117,118]
BM-MSCs	Exos	cardiac stem cells/in vitro/ischemia/reperfusion injury	prevention of apoptosis and increased survival and proliferation	miR-15, miR-21, miR-22, miR-126, miR-146a, miR-210	[119,120]
BM-MSCs	Exos	cardiomyocytes/macrophages/dilated cardiomyopathy	reduced apoptosis of cardiomyocytes/reduced production of inflammatory cytokines in macrophages//improved myocardial function/attenuated cardiac dilation	IL-10/VEGF	[121]

Abbreviations: bone marrow (BM); mesenchymal stem cells (MSCs); exosomes (Exos); phosphoinositide-3-kinase (PI3K); protein kinase B (Akt); AMP-activated protein kinase (AMPK), mammalian target of rapamycin (mTOR); microRNA (miR); stromal cell-derived factor-1 (SDF-1); interleukin (IL)-10; vascular endothelial growth factor (VEGF).

**Table 6:** Therapeutic potential of MSC-derived secretome in the therapy of eye disease.

Source Cell Type	Type of Secretome	Target Cells/Condition	Effects/Major Findings	Pathways Involved	Ref. No.
BM-MSCs	Exos	RGCs/glaucoma	Increased survival and regeneration of RGCs/attenuation of glaucoma	BDNF, NGF, PDGF, miR-17-92, miR-21 and miR146a	[122,123,124]
BM-MSCs	Exos	macrophages/laser-induced retinal injury	attenuated activation of inflammatory macrophages/alleviation of retinal inflammation/increased number of photoreceptor cells	MCP-1	[34]
BM-MSCs	Exos	neutrophils, NK cells, macrophages and T cells/EAU	reduced influx of inflammatory cells/attenuation of EAU	MCP-1; CCl21	[35]
BM-MSCs	Exos	DCs, Th1, Th17 cells/EAU	attenuation of antigen-presenting function of DCs; reduced production of Th1 and Th17-related cytokines/attenuation of EAU	IL-10; IDO-1/KYN	[125]
BM-MSCs	CM/Exos	macrophages; Th1 and Th17 cells/corneal injury; DED	reduced production of IL-1β/attenuated activation of Th1/Th17 cells/alleviated corneal inflammation	IL-1Ra; GRO; IOD-1/KYN	[126,127,128,129,130]
UCD-MSCs	Exos	keratocytes/Sly syndrome	enhanced degradation of GAGs/attenuation of Sly syndrome	β-glucuronidase-induced degradation of GAGs	[132]

Abbreviations: bone marrow (BM); umbilical cord (UCD); mesenchymal stem cells (MSCs); exosomes (Exos); retinal ganglion cells (RGCs); brain-derived neurotrophic factor (BDNF); nerve growth factor (NGF); platelet-derived growth factor (PDGF); experimental autoimmune uveitis (EAU); monocyte chemoattractant protein 1 (MCP-1); Chemokine (C-C motif) ligand 21 (CCL21); natural killer (NK) cells; dendritic cells (DCs); interleukin (IL)-10; indoleamine 2,3 dioxygenase-1 (IDO-1); Kynurenine (KYN); dry eye disease (DED); IL-1 receptor antagonist (IL-1Ra); and growth related oncogene (GRO); glycosaminoglycans (GAGs).

**Table 7:** MSC-derived secretomes in the therapy of ischemic brain damage and spinal cord injury.

Source Cell Type	Type of Secretome	Target Cells/Condition	Effects/Major Findings	Pathways Involved	Ref. No.
BM-MSCs	Exos	neurons/ischemic brain injury	improved neurogenesis and neurite remodeling	miR-124 and miR-133b	[36]
BM-MSCs	Exos	neurons/SCI	enhanced regeneration of axons	miR-133b/Erk1/2 and Stat-3	[134]
BM-MSCs	Exos	N1 astrocytes/SCI	suppressed production of inflammatory cytokines	IL-10/NF-kB-p65	[135,137]
BM-MSCs	Exos	macrophages/SCI	conversion from inflammatory M1 to immunosuppressive M2 phenotype	IL-10/NF-kB-p65	[138]
UCD-MSCs	Exos	neurons; glial cells/nerve-injury induced pain	reduced excitation of neurons, activation of glial cells/attenuation of nerve-injury induced pain	BDNF, GDNF/IL-10	[139]
BM-MSCs	EVs	pericytes/SCI	reduced migratory capacities of pericytes/increased integrity of BSCB/improved motor function	NF-kB-p65	[140]

Abbreviations: bone marrow (BM); mesenchymal stem cells (MSCs); exosomes (Exos); extracellular vesicles (EVs); umbilical cord (UCD); interleukin (IL)-10; spinal cord injury (SCI); nuclear factor-kB (NF-kB); brain-derived neurotrophic factor (BDNF); glial cell line-derived neurotrophic factor (GDNF); blood-spinal cord barrier (BSCB).

## Abbreviations

1-MT	1-methyl-dl-tryptophan
ALF	acute liver failure
ALI	acute lung injury
AT-MSCs	adipose tissue-derived MSCs
bFGF	basic fibroblast growth factor
BSCB	blood-spinal cord barrier
HSP	heat-shock proteins
BDNF	brain-derived neurotrophic factor
CSCs	cardiac stem cells
Cav-1	caveolin-1
COPD	chronic obstructive pulmonary diseases
CD	Crohn’s disease
CMV	cytomegalovirus
CTLs	cytotoxic T lymphocytes
DSS	dextran sodium sulphate
DED	dry eye disease
ECs	endothelial cells
EGF	epidermal growth factor
EGFR	epidermal growth factor receptor
Exos	exosomes
EAU	experimental autoimmune uveitis
EVs	extracellular vesicles
Fap-1	Fas-associated phosphatase-1
FGF-6	fibroblast growth factor 6
GCN2	general control nonderepressible 2
GDNF	glial cell line-derived neurotrophic factor
GSH	glutathione
GAGs	glycosaminoglycans
GRO	growth related oncogene
HGF	hepatic growth factor
HSCs	hepatic stellate cells
HSV	herpes simplex virus
hAT-MSC-CM	human adipose tissue MSC-derived conditioned medium
hUTC-MSC-CM	human uterine cervical MSC-derived conditioned medium
HIF-1α	hypoxia-inducible factor 1 alpha
IPF	idiopathic pulmonary fibrosis
IL-1Ra	IL-1 receptor antagonist
IDO	indolamine 2-3-dioxygenase
iNOS	inducible nitric oxide synthase
IBDs	inflammatory bowel diseases
KGF	keratinocyte growth factor
KYN	kynurenine
MMIF	macrophage migration inhibitory factor
MHC	major histocompatibility complex
MDA	malondialdehyde
mTOR	mammalian target of rapamycin
MMP	matrix metalloproteinase
MSCs	mesenchymal stem cells
MCP-1	monocyte chemotactic protein-1
MSC-CM	MSC-derived conditioned medium
MTF	MSC-derived trophic factors
MPO	Myeloperoxidase
NSF	N-ethylmaleimide-sensitive factor
NK	natural killer
NKT	natural killer T
NGF	nerve growth factor
nSMase2	neutral sphingomyelinase 2
NO	nitric oxide
NF-κB	nuclear factor κB
OA	osteoarthritis
OVA	ovalbumin
PB-MNCs	peripheral blood mononuclear cells
PTEN	phosphatase and tensin homolog
PGF	placental growth factor
PDGF	platelet-derived growth factor
PGE2	prostaglandin E2
RhoA	Ras homolog gene family member A
RGCs	retinal ganglion cells
S1P	sphingosine 1-phosphate
SCI	spinal cord injury
SOD	superoxide dismutase
TSP1	thrombospondin 1
TIMP-1	tissue inhibitors of metalloproteinase
TGF-β	transforming growth factor-β
Tregs	T regulatory cells
TNFSF14	tumor necrosis factor superfamily member 14
UC	ulcerative colitis
VEGF	vascular endothelial growth factor
